# Ultra-Low Frequency Eccentric Pendulum-Based Electromagnetic Vibrational Energy Harvester

**DOI:** 10.3390/mi11111009

**Published:** 2020-11-16

**Authors:** Mingxue Li, Huichao Deng, Yufeng Zhang, Kexin Li, Shijie Huang, Xiaowei Liu

**Affiliations:** 1 MEMS Center, Harbin Institute of Technology, Harbin 150001, China; 16b921022@stu.hit.edu.cn (M.L.); yufeng_zhang@hit.edu.cn (Y.Z.); 19S121097@stu.hit.edu.cn (K.L.); 1173200517@stu.hit.edu.cn (S.H.); akula.zhong@gmail.com (X.L.); 2 School of Mechanical Engineering and Automation, Beihang University, Beijing 100191, China

**Keywords:** eccentric pendulum, ultra-low frequency, vibration energy harvesting

## Abstract

With the development of low-power technology in electronic devices, the wireless sensor network shows great potential in applications in health tracing and ocean monitoring. These scenarios usually contain abundant low-frequency vibration energy, which can be collected through appropriate energy conversion architecture; thus, the common issue of limited battery life in wireless sensor devices could be solved. Traditional energy-converting structures such as the cantilever-beam type or spring-mass type have the problem of high working frequency. In this work, an eccentric pendulum-based electromagnetic vibration energy harvester is designed, analyzed, and verified with the finite element analysis method. The pendulum that contains alternative distributed magnets in the outer side works as a rotor and has the advantages of a simple structure and low center frequency. The structure size is well scalable, and the optimal output performance can be obtained by optimizing the coil thickness and width for a given diameter of the energy harvester. The simulation results show that the energy harvester could work in ultra-low frequencies of 0.2–3.0 Hz. A full-scale prototype of the energy harvester is manufactured and tested. The center working frequency is 2.0 Hz with an average output power of 8.37 mW, which has potential for application in driving low-power wireless sensor nodes.

## 1. Introduction

The wireless sensor network (WSN) could monitor environmental parameters with advantages of high efficiency and total automation, and it has great application potential for many aspects [1,2]. Especially in fields of wireless body area network (WBAN) for personal health monitoring [3,4,5] and wireless oceanic sensor network (WOSN) [6,7,8], the WSN system benefits from its unmanned-working feature to take full advantage of high promptness and give a warning of anomalous environment or body status change in time. However, the limited battery lifetime has always been a severe issue, which blocks large-scale applications of WSN [9,10]. The sensor network usually has numerous nodes that spread over a large area, and it takes a lot of work to change or charge batteries for each node, no matter whether the node is powered by a primary battery or secondary battery. In addition, the abandon of large amounts of batteries will cause serious environmental pollution [11,12,13]. As a result, the investigation of a power supply system that contains renewable energy has attracted many scholars′ attention, and it is a new trend to introduce multiple renewable energy types and converting mechanisms for improving performance [14,15]. It is a feasible method to solve the problem of wireless-sensor power source.

Common environmental energy sources that wireless sensor nodes could harvest and convert to electric power include solar energy [16,17,18], wind energy [19,20,21], radio-frequency (RF) energy [22,23,24], and vibration mechanical energy [25,26,27]. The photovoltaic cell, as an energy-harvesting device for solar energy with high efficiency and mature commercialization, has been widely used in the field of self-powered sensor system. However, solar energy in the environment is easily affected by illumination and weather, which makes it unstable [28]. In the indoor environment without direct sunlight, the efficiency of solar cells will be greatly reduced [29]. These problems could also occur with small wind turbines. The RF energy is a form of energy that always exists in space, but its power density is very low, which makes it difficult to drive the wireless sensor devices directly [30]. The vibrational mechanical energy is very common in outdoor or indoor environments and has relative high-power density, especially in marine environments [31]. Thus, the vibration energy harvester is a befitting self-sustaining device for low-power wireless sensor nodes.

The vibration amplitude and frequency are quite various and random in different environments. For the vibration excited by ocean waves or the body movements of humans, it has characteristics of low frequency and high swinging amplitude [32,33], which indicates that the energy harvester should have a low working frequency and wide frequency range to harvest the vibration energy in these scenes. According to the working principle of a transducer, the types of vibrational energy harvester can be divided into electrostatic, piezoelectric, and electromagnetic [34]. Based on the principle of variable capacitance and micro-electro-mechanical system (MEMS) technology, the electrostatic vibration energy harvester usually operates at a frequency higher than 100 Hz [35,36,37]. The piezoelectric-based vibrational energy harvester typically introduces a cantilever with piezo film as the transducer, and the working frequency is usually lower than electrostatic types [38,39,40]. The center frequency could be further decreased by introducing converting structures such as magnetic plucking [41,42]. Thus, to utilize an electrostatic or piezoelectric-based energy harvester to scavenge the low-frequency vibration energy, it is essential to use a complex frequency lifting structure; however, designing and manufacturing such a structure is arduous. The electromagnetic vibration energy harvester is more flexible in structure and could respond to ultra-low frequency vibration via reasonable design. For example, the non-linear planar spring structure could be manufactured via a high precision machining process, which makes it possible for frequency decreasing and bandwidth spreading using a multi-modal non-linear spring. Roy et al. utilized fiberglass-epoxy board with particular patterns and a pair of repulsive magnets on the end to form a non-linear bistable planar spring [43]. The resonant frequency was reduced from 55.4 to 29.5 Hz. Chen et al. combined a MEMS coil and U-shape cantilever and fabricated a micro dual-modal electromagnetic vibration energy harvester with double working frequencies of 211 Hz and 274 Hz [44]. Using a magnetic levitation effect could avoid a complex spring structure design and lower the frequency more efficiently. Meanwhile, a movable magnet could work as a mass block directly, which makes the harvester more compact. Jo et al. used multilayer polyimide-based planar coils and a magnetic repulsive structure to fabricate a miniature energy harvester [45]. The working bandwidth is 1–15 Hz and the center frequency is 8 Hz. Ulusan et al. used the non-linear magnetic-levitation spring to form a cylindrical solenoid vibration energy harvester with the working frequency of 15 Hz [46]. Riduan et al. decreased the volume of this structure to the traditional AA-size battery, which was easy to be mounted in an electronic devices [47]. Geisler et al. increased the poles of magnets and coils to enhance the power performance based on the AA-size cylinder and decreased the frequency to 6 Hz [48]. To further decrease the center frequency to adapt to an ultra-low-frequency vibration environment, a free oscillating magnetic mass block structure is used, where the spring serves only as a buffer or even there is no spring. Bendame et al. introduced a sliding rail and free-sliding magnet to realize the bandwidth of 6 Hz [49]. Fan et al. used two loop springs as the suspension structures of a magnetic rotor and mass tip, respectively, and introduced different twisting angles between the springs [50]. The harvester could work in the frequency range of 1.8–2.6 Hz with 12.3 mW output power. Gu et al. designed a goblet-like oscillating cavity with bistable sliding chutes for magnetic balls and realized 10 Hz bandwidth and low working frequency [51]. The rolling magnets work as a sliding mass tip that has lower mechanical damping. Xu et al. investigated a pendulum-based cantilever energy harvester and realized few-Hertz multi-direction vibration energy harvesting [52]. Fu et al. introduced a bi-stability rotary-translational-motion energy-converting structure and realized energy harvesting at an ultra-low frequency and low acceleration [53]. With the excitation of 0.8 Hz and acceleration of 0.6 m/s^2^, the energy harvester reached a root-mean-square output power of 60 µW.

Based on the principle of free rotating motion, a circular or sector eccentric-pendulum structure could achieve a lower working frequency and thus attracted some focus. Pit et al. aimed at the kinetic energy of a human movement collection and introduced a half-circle eccentric pendulum as the frequency-lifting structure to drive the cantilever-based piezoelectric vibration energy harvester [54]. The designed harvester reached a peak output power of 43 µW with an acceleration of 2 g and frequency of 2 Hz. Reza et al. employed a ring-shaped eccentric pendulum instead of a half-circle shape to accommodate more numbers of magnetic poles and reach a higher trigger frequency of the piezoelectric beam [55]. With a 150 g mass tip, 0.5 g acceleration, and 2 Hz vibration frequency, the energy harvester reached the best performance of 0.4 mW. Byung-Chul et al. designed a compound double pendulum structure that contained a pendulum of magnets and a pendulum of coils, which could realize anti-phase motion [56]. The harvester generated the best output power of 247 µW at the vibration frequency of 2 Hz and the root-mean-square voltage reached around 0.6 V. As a result, the pendulum-based energy harvester is well fit for power conversion from ultra-low-frequency vibration. However, there is still a lot of room for improvement in the simplification of the structural design and the enhancement of output power.

According to the analysis above, it could be concluded that an electromagnetic vibration energy harvester could effectively convert the low-frequency vibration energy to electric energy. However, there are still few investigations realizing a vibration energy transducer of ultra-low frequency. In this work, a free-swing eccentric pendulum-based magnetic vibration energy harvester with wide bandwidth and ultra-low frequency is designed. The volume of the energy harvester is compact, and the structure is simple. All parts of the energy harvester are easy to be manufactured, and the assembly process is very simple. As the generating components are based on electromagnetic energy, the output power is relatively high, and the voltage range is moderate. The vibration energy harvester could work in the ocean wave or human motion environment and supply electric power for wireless sensor systems.

## 2. Structure Design and Analysis Model of the Energy Harvester

**Figure 1:**
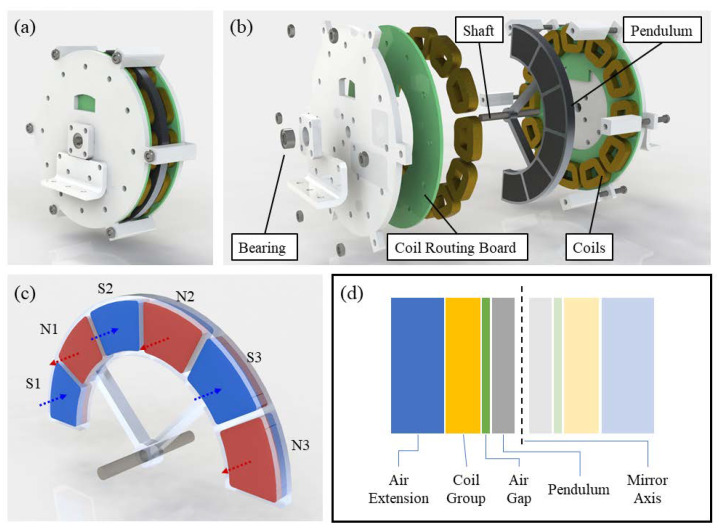
Structure design of a low-frequency vibration energy harvester: (**a**) assembly diagram of an energy harvester; (**b**) explosive view of an energy harvester; (**c**) magnetic field configuration for the eccentric rotor; (**d**) framework diagram of the finite element analysis (FEA) model.

As the available space for power sources in a typical WSN node system is usually limited, the structural volume and complexity of the energy harvester should be as small as possible. In this work, we proposed a novel electromagnetic generator structure where the magnetic field is parallel to the rotation axis, and we introduced an eccentric disk with multiple groups of permanent magnets as the rotor, which is shown in Figure 1a. The structural decomposition diagram of the energy harvester is shown in Figure 1b. Two sets of coils are symmetrically distributed on both sides of the eccentric pendulum, and each set of coils is parallel to the planar surface of the rotor. The coils of the same side are mounted on a printed circuit board (PCB) and are connected in series via the routes within the PCB. The shaft is made of 304 L stainless steel and fixed to the housing of the energy harvester with bearings at both ends, which reduces the rotational friction damping of the rotor effectively. The magnetic-field configuration of the energy harvester is shown in Figure 1c. Each of the two adjacent permanent magnets has an opposite magnetic-field direction and forms three groups of polar pairs. Thus, an alternating magnetic field is formed normal to the pendulum. The magnets are made of Nd-Fe-B alloy, whose brand is N35, and they are fixed into the pendulum framework with glue or screws. The pendulum frame is manufactured using aluminum alloy, which causes the material density of the outer side to be much lower than the inner side. The inner side of the pendulum frame only retains the beam used for structural support, which further reduces the equivalent density of the inner side. Under this design structure, the permanent magnet set is used as the magnetic field generation component of the electromagnetic generator and also as the eccentric mass block component of the vibration energy conversion structure, which has the advantage of being a simple and compact structure. The horizontal layout of the coils makes the overall thickness of the energy harvester smaller and makes the horizontal scalability enhanced. For example, if the space is abundant, the number of pendulum rotors and coil layers could be increased to improve the output power exponentially. There is no mechanical position limitation to the rotor, and the mechanical damping of the rotor is very low, so that the pendulum could swing with high sensitivity to the external vibration, and the eccentric rotor has a very low center frequency due to the radical shift of the centroid.

According to the structural design of vibrational energy harvester mentioned above, the finite element analysis (FEA) method is introduced to study the power-generating mechanism and output performance of the energy harvester, and the architecture of the FEA model is shown in Figure 1d. Since the whole energy harvester is symmetrical with respect to the median plane, the FEA model only needs to contain the structure of a single side to reduce the complexity of the calculation. The boundary condition of the median plane is configured as a mirror symmetric to realize the simulation of the whole generator. The half-cut eccentric pendulum layer adopts the geometric structure as shown in Figure 1c, which works as the magnetic field source. A thin layer of air is set between the coil layer and pendulum layer. On the other side of coil layer, an excess layer of air is used to simulate the dispersion of the magnetic field. The air-gap layer is separated into two thin layers with equal thickness. The thin layer near the pendulum forms the rotor region along with the eccentric pendulum layer, and the other thin layer forms the stator region together with the coil layer and air-extension layer.

The overall governing equations of the model include Maxwell’s equations for electromagnetic field simulation and multi-body dynamic equations for motion simulation. The core problem of electromagnetic-field calculation lies in the solution of magnetic-potential distribution in the energy harvester. For the rotor region without current conduction, the calculating complexity could be reduced by utilizing the scalar magnetic potential, $\varphi_{m}$. The magnetic field strength equation could be expressed as

(1) ∇⋅B=0,

(2) $$H=-\nabla \varphi_{m}$$

where B is the magnetic flux density and H is the magnetic field intensity. For the air and eccentric pendulum parts, the relation equation of B and H is

(3) $$B=\mu_{0}\mu_{r}H,$$

where $\mu_{0}$ is the permeability of vacuum and $\mu_{r}$ is relative permeability. For the parts of permanent magnets, the residual flux density $B_{r}$ is added to the relation equation, which is expressed as

(4) $$B=\mu_{0}\mu_{r}H+B_{r},.$$

The scalar magnetic potential could be solved by simultaneous equations of (1)–(4). The vector magnetic potential A is introduced to describe the electromagnetic-field distribution and the equations are

(5) ∇×H=J

(6) B=∇×A,

(7) $$E=-\frac{\partial A}{\partial t},$$

where J is current density, E is electric field intensity, and t is time. The relation equation of B and H could still be satisfied with Equation (3). The current density exists in the coil region and could be calculated by

(8) $$J=\frac{NI_{L}}{A}\cdot e_{coil},$$

where N stands for the turns of the coil, $I_{L}$ stands for the load current, and $e_{coil}$ stands for the direction of the infinitesimal element in the coil. When the energy harvester is in the external vibrational excitation, the motion equation of the rotor could be expressed as

(9) $$g\sin\theta +a_{ext}\sin\left(2\pi ft+\phi_{0}\right)\cos\theta +c_{m}\dot{\theta }+I\ddot{\theta }+F_{mag}=0,$$

where g is the gravitational acceleration, θ is the rotating angle of the rotor, $a_{ext}$ is the acceleration of external excitation, f is the frequency of external excitation, $\phi_{0}$ is initial phase of external excitation, $c_{m}$ is the mechanical damping coefficient, I is the moment inertia of the pendulum, and $F_{mag}$ is the damping force caused by the electromagnetic field, which could be calculated by volume integration of the simulating element’s electromagnetic torque, whose expression is

(10) $$F_{mag}=\int {\;}_{\Delta \Omega }nT_{mag}dS,$$

where $T_{mag}$ is the Maxwell’s electromagnetic stress tensor of the calculating element and ΔΩ stands for the surface enclosed by the calculating element.

In the FEA model, the Arbitrary–Lagrange–Euler (ALE) method is introduced to describe the rotation of the rotor, and a continuity equation is used at the interface between the rotor and stator to ensure the correct transfer of physical field parameters. The whole FEA model of the energy harvester could move under the external low-frequency oscillating excitation. Under such excitation of external vibration, the rotating status of the eccentric rotor is transmitted to the electromagnetic field model. Thus, the relative motion between the permanent magnets and the coils, as well as the voltage and current characteristics of the coil output, can be calculated. To verify the load-driven ability of the energy harvester, the terminal of the coil group is connected to the electric circuit (EC) field as a current source. A load resistor is connected between the output port of the energy harvester and the reference ground to calculate the output power.

## 3. Results and Discussion

### 3.1. Analysis of Magnetic Field Distribution and Power Generation Mechanism

For the electromagnetic vibration energy harvester, the distribution of the magnetic field is one of the most important factors to determine its power generation effect. In the meantime, the mechanism of power generation in the energy harvester could be described by analyzing the pattern of relative motion between the magnets and coils. Thus, we conduct the simulation of the magnetic field when the energy harvester is in the steady state and motion state separately at first.

**Figure 2:**
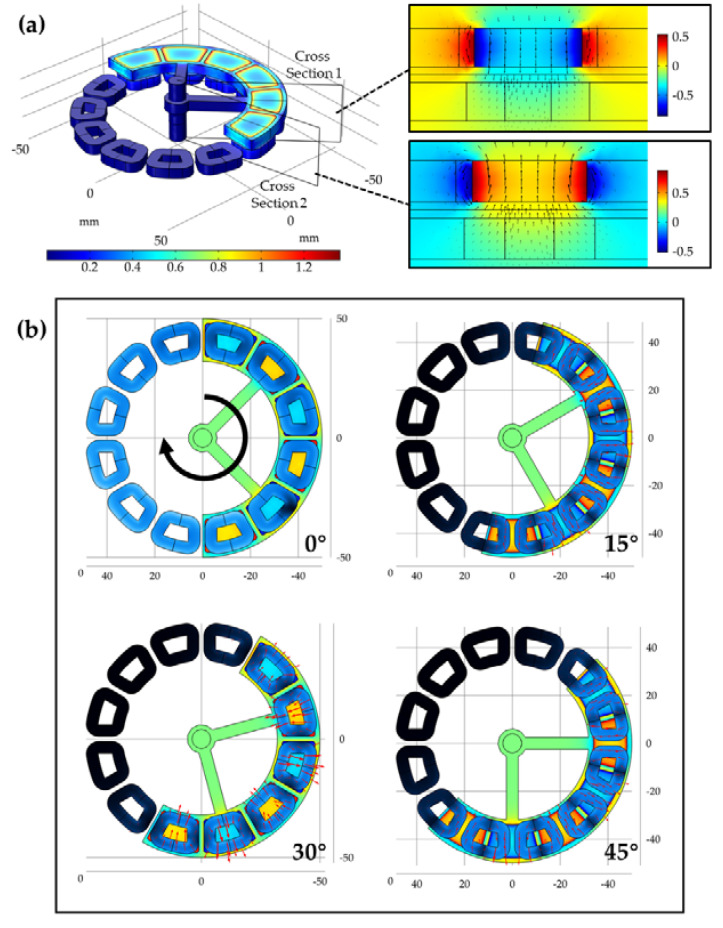
(**a**) Magnetic flux density distribution results; (**b**) the z-component of magnetic flux density distribution results on the cross-sections of the adjacent magnets.

The spatial distribution of the magnetic field intensity could be expressed by magnetic flux density, which is shown in Figure 2a. The strongest magnetic flux density lies in the permanent magnets of the rotor, and the magnetic field diffuses through the air gap to the coil layer of the stator. In the three-dimensional distribution results on the left of Figure 2a, we cut the planes in the middle of two adjacent permanent magnets respectively, so that the magnetic field transfer from the permanent magnets to the coils could be observed more intuitively. The distribution data in both subgraphs stand for the z-component of magnetic flux density, and the black arrows indicate the directions of the magnetic field. The length of the arrow is determined by the strength of the magnetic field. In the center of the magnets, the direction of the magnetic field is along z-axis. In the marginal area, the magnetic field bends to the opposite direction. As a result, there are vertical magnetic-induction lines going through the area surrounded by coils, and magnetic fields of adjacent coils are always in opposite directions.

The alternatively distributed effect magnetic flux is generated in the coils that are covered by rotor magnets according to the three-dimensional static analysis of the energy harvester. When the pendulum begins swinging due to external vibration, the magnetic flux in the coils will change, and the induced electric field will be generated. The electric field distribution in the coils is simulated and analyzed when the rotor is spinning. In this model, the rotor is forced to rotate at a certain constant angular velocity, and the direction is clockwise. The simulation results of different angles that the rotor experiences within a complete generation cycle are shown in Figure 2b. At the initial state when the rotation angle is 0°, the center of coils that are covered by the rotor coincides with the center of the magnets. When the rotor turns clockwise and the rotation angle is less than 30°, the two radial-side arms of the middle coils are covered by the magnetic field with opposite poles due to the alternating placement of magnet poles. Each side arms the magnetic induction line along the angle direction to form an opposite electric field, and the clockwise loop is formed in these coils. For the marginal coils that the rotor enters and leaves, only one side arm generates an inductive electric field and produces half as much power as the middle coils. When the rotation angle reaches 30°, the coils are covered with magnets whose pole directions are opposite to the initial state, and both side arms generate an electric field with same direction, which causes neutralizing and the output voltage to drop to 0 V. When the rotor continues to rotate and the rotation angle is less than 60°, the electric field of the opposite direction begins to be generated in the coil, forming the negative half cycle of the output voltage. When the rotation angle reaches 60°, the coil coverage state returns to the initial state, and a generation cycle is completed.

### 3.2. Optimization of Coil Design Parameters

**Figure 3:**
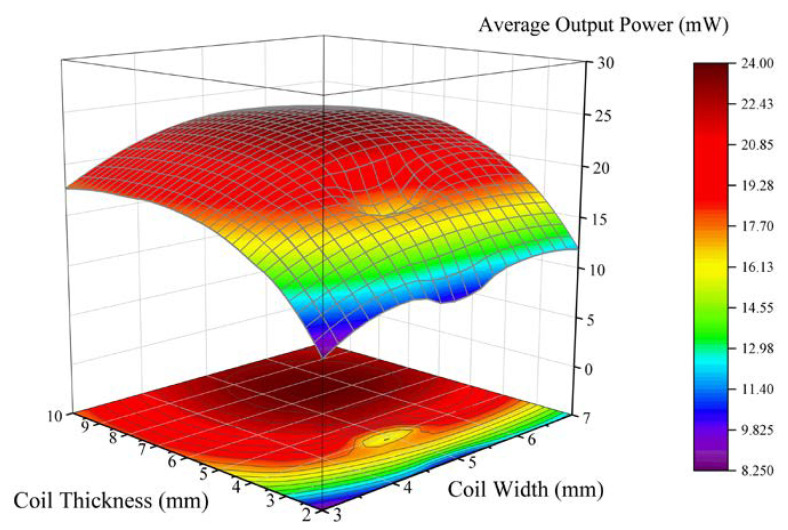
Output power of the energy harvester under different combines of coil width and thickness.

In the structure of the energy harvester, the rotor magnetic field is transmitted to the stator coil through the air gap. Since the intensity of the magnetic field transferred in the air is related to the distance between the magnets and coils, the effective magnetic flux of each layer of coil with a certain thickness is different. Although the number of coil turns that participate in power generating will increase with the increasing of coil thickness, the impedance of the coil also increases, and the power generation capacity of the bottom coil is less than that of the top coil. As a result, there is an optimal designing value for the coil thickness to maximize the output efficiency of the coil. For the width of the coil, the best equivalent generating length can be obtained if the contour outside the coil coincides with the magnet geometry. More turns could be obtained by winding inwards, but the effective generating length also decreases and the coil impedance increases, which might lead to generating performance reduction. Therefore, the coil width is also one of the important parameters for performance optimization. For these two geometric parameters, we select multiple combinations of both parameters in the FEA model and calculate the average output power of the coil. In all these models, the rotor speed is set at a constant value of 1 rps to compare the performance variation. The output power results are shown in Figure 3. The X-axis and Y-axis represent the width and thickness of the coil respectively, and the Z-axis represents the average output power of the corresponding width and thickness. As it could be observed from the rainbow map, the output power presents a peak shape, indicating that there is a binary optimal value for the coil design parameters. The optimal design width and design thickness of the coil are 6 mm and 7 mm respectively. This optimization method is applicable to a vibration energy harvester of any size.

### 3.3. Low-Frequency Vibrational Energy Harvesting Test

**Figure 4:**
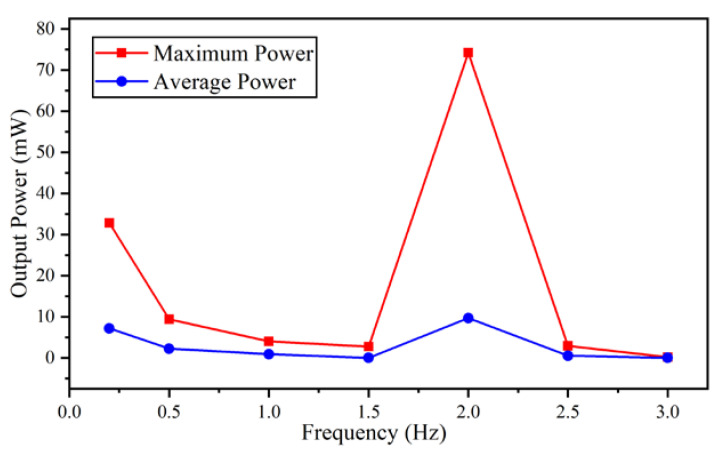
The energy harvester output results under low frequency vibration excitation.

By means of the ALE method, the external low-frequency vibration excitation could be applied to the vibration energy harvester in the finite element analysis model. To verify the energy harvesting effect from low-frequency vibration, the vibrational environment is added into the energy harvester model, and the real-time output waveform of the energy harvester is calculated. The waveform of the vibrational excitation is sinusoidal and its frequency ranges from 0.2 to 3 Hz. The amplitudes of acceleration are 0.1 g. The simulation results of the maximum output power and average output power under these test conditions are shown in Figure 4. As the output voltage is in alternative-current (AC) form, there are values of peak power and mean power in power calculation. The average power is obtained by summing the output power over a period of time and dividing it by the length of time. The maximum peak power is 74.21 mW, and the average power is 9.69 mW when the frequency of vibration excitation is 2 Hz, which shows that the resonant frequency of the vibration energy harvester is near this frequency point. With the increasing or decreasing of excitation frequency, the output performance begins dropping. However, when the excitation frequency drops to 0.2 Hz, another peak power point appears. The average output power when the frequency is 1.5, 2.5, and 3.0 Hz is 2.76, 2.921, and 1.854 mW, respectively. These simulation results indicate that the vibrational energy harvester could convert mechanic energy into electric energy when the vibration frequency is very low. Even the frequency offsets from the center frequency, the energy harvester could still supply the electric power of milliwatt magnitude, which is suitable for harvesting energy of environmental ultra-low frequency vibration with some tolerance of randomness.

### 3.4. Vibrational Energy Harvesting Test Using Prototype

**Figure 5:**
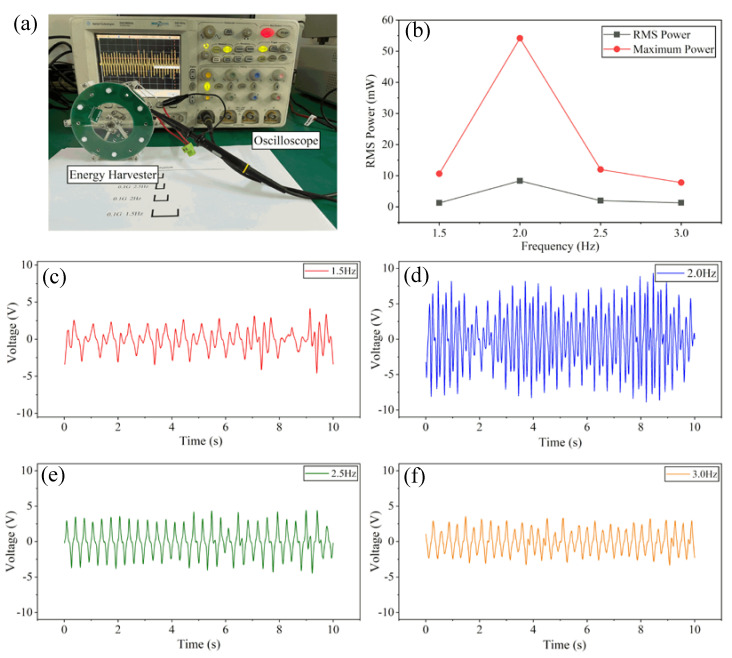
(**a**) Photo of the test platform; (**b**) root-mean-square and maximum output power results of the prototype; (**c**) output voltage waveform under 1.5 Hz vibration; (**d**) output voltage waveform under 2.0 Hz vibration; (**e**) output voltage waveform under 2.5 Hz vibration; (**f**) output voltage waveform under 3.0 Hz vibration.

In order to verify the structural function of low-frequency vibration energy harvesting, we made a prototype based on the optimization results of by the finite element analysis model as described above. The test platform and the prototype photos are shown in Figure 5a. The manufacturing parameters of the prototype are listed in Table 1. 

The vibrational excitation is generated by a shaker that is driven by a reciprocal motor. The consistency of acceleration could be guaranteed by adjusting motor’s stroke length and oscillation frequency. The vibration frequencies are 1.5, 2.0, 2.5, and 3.0 Hz, and the vibration acceleration is 0.1 g. The output voltage waveform of the energy harvester is captured in real time by an oscilloscope, whose model is DSO6054A. The voltage waveforms when the energy harvester is under the vibration frequency of 1.5, 2.0, 2.5, and 3.0 Hz are shown in Figure 5c–f respectively. The output voltage waveforms are the overlay of sinusoidal waves of different frequencies, which is the result of the simple harmonic swinging motion of the eccentric pendulum in the process of low-frequency vibration. When the pendulum swings to a certain height, the rotating velocity drops to zero and then rotates in a reverse direction with acceleration, resulting in a non-linear change rate of the magnetic flux in the coils. When the vibration frequency is 2.0 Hz, the output voltage and the alternative frequency reached peak values, which stand for the center frequency of the energy harvester. The maximum output power and root-mean-square (RMS) output power of different excitation frequencies are shown in Figure 5b. Consistent with the simulation results, the output power of the energy collector reaches the maximum at the frequency of 2 Hz, and the RMS power and maximum power are 8.37 and 54.2 mW respectively. Under the vibration frequency of 1.5, 2.5, and 3.0 Hz, the RMS values of output power are 1.27, 2.00, and 1.32 mW, respectively, while the maximum values are 10.66, 12.02, and 7.82 mW, respectively. When the vibration frequency deviates from the central frequency, the output power of the energy harvester decreases obviously, but its power level can still meet the requirements of some low-power-consumption working mode of the wireless sensor node system such as the processor running at low frequency, driving sensors, system standby, and so on.

**Figure 6:**
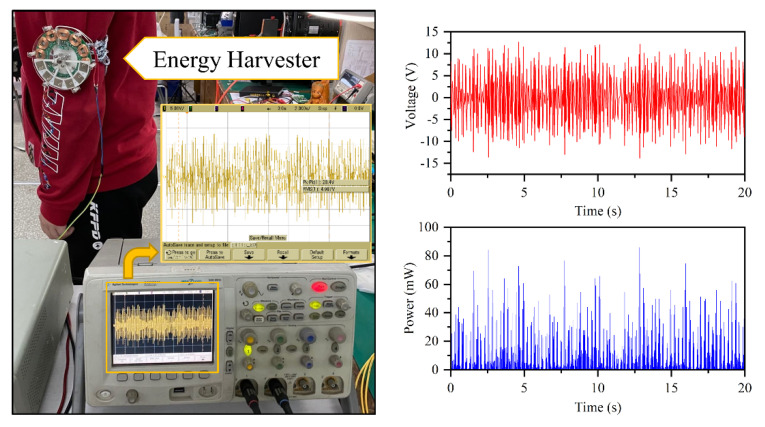
Application demonstration results of human kinetic energy harvesting using the energy harvester: output voltage wave and instantaneous power.

When the human is walking, a typical low-frequency vibration environment is formed on the limbs. In this work, we demonstrated the application of the energy harvester to convert human-body kinetic energy to electric energy. The prototype was fastened to the upper arm, and the pendulum rotated when the people to be tested began walking. The output port is connected to the oscilloscope to record the real-time waveform and the effective value of voltage, which is shown in Figure 6. The RMS voltage is 4.987 V, and the average generating power is 11.3 mW. The maximum instantaneous voltage and power reached 13.75 V and 85.9 mW, respectively. The higher output power indicates that the vibration intensity produced by human walking is high, and the energy harvester will be suitable for low-power-consumption wearable electronic devices.

The best working condition for the energy harvester designed in this work is 2 Hz vibrational excitation. However, in many other scenes, the mechanical energy is contained in a vibration of lower frequency, which could cause the decreasing of energy harvesting effect. To overcome this situation, the gravity torque should be extended by way of increasing the effective mass of magnets and the radius of the eccentric rotor. The randomness of vibration in the environment will also affect the power generation effect of the energy harvester, so the bandwidth expansion is also very important. This could be realized by adding the non-linear degree of the energy conversion mechanism, such as an adopting energy harvester array that consists of cells with different moment of inertia or employing a non-linear spring. In the future work, we will further decrease the center frequency of the energy harvester and expand the effect bandwidth to develop its application potential in harvesting the vibration energy of water wave.

## 4. Conclusions

In this work, an eccentric pendulum-based magnetic vibrational energy harvester is designed. The springless structure makes it possible for ultra-low-frequency vibrational energy harvesting. Alternating magnets on the pendulum create alternating magnetic fields in the coils on either side, which drive the coils to generate electricity with the rotor swinging excited by external vibration. The magnetic-field arrangement of the electromagnetic generator makes the energy harvester simple in structure and flexible in size. With the help of the FEA method, the output performance of the energy harvester could be evaluated. When the overall size of the energy harvester is determined, the most optimal values for the design parameters of coil width and thickness are found. To verify the effect of collecting low-frequency vibration energy, the output power performance is simulated when the energy harvester is excited by the low-frequency vibration ranging from 0.2 to 3 Hz with the peak acceleration of 0.1 g. The vibrational energy harvester reaches the best performance when the frequency of vibration is 2 Hz, and in this case, the average output power is 9.69 mW and the maximum output power is 74.21 mW. The results show that the central frequency point of the vibration energy harvester is very low and the sensitivity of the energy harvester is high, which is suitable for a low-frequency vibration environment. In the end, a full-scale prototype of the energy harvester is manufactured, fabricated, and tested under the 0.1 g acceleration with frequency domain from 1.5 to 3 Hz. The results prove that the center frequency of the energy harvester is 2.0 Hz, and the RMS power is 8.37 mW, which is basically consistent with the simulation results of the FEA model. The simulation and prototype results show that the designed energy harvester has potential in ultra-low-frequency vibration situations such as human wearables, ocean waves, and so on. The output power is sufficient to drive low power processors, sensors, etc.

## Figures and Tables

**Table 1:** Design parameters of the prototype.

Name of Component	Properties	Parameters
Pendulum	Framework Material	ABS resin
	Radius	50 mm
	Magnet Material	Nd-Fe-B Alloy
	Magnet Brand	N35
Coil	Inner Radius	33.5 mm
	Outer Radius	48.5 mm
	Sector Angle	30°
Shaft	Diameter	4 mm
	Material	Stainless Steel
Overall Size		Φ100 mm × 19.6 mm

## References

[1] Ojha T., Misra S., Raghuwanshi N.S. (2015). Wireless sensor networks for agriculture: The state-of-the-art in practice and future challenges. Comput. Electron. Agric..

[2] Ali A., Ming Y., Chakraborty S., Iram S. (2017). A Comprehensive Survey on Real-Time Applications of WSN. Future Internet.

[3] Latré B., Braem B., Moerman I., Blondia C., Demeester P. (2017). A Survey on Secure Wireless Body Area Networks. Secur. Commun. Netw..

[4] Ha I. (2015). Technologies and Research Trends in Wireless Body Area Networks for Healthcare: A Systematic Literature Review. Int. J. Distrib. Sens. Netw..

[5] Hao Y., Foster R. (2008). Wireless body sensor networks for health-monitoring applications. Physiol. Meas..

[6] Shahanaghi A., Yang Y.L., Buehrer R.M. (2020). Stochastic Link Modeling of Static Wireless Sensor Networks Over the Ocean Surface. IEEE Trans. Wirel. Commun..

[7] Felemban E., Shaikh F.K., Qureshi U.M., Sheikh A.A., Qaisar S.B. (2015). Underwater Sensor Network Applications: A Comprehensive Survey. Int. J. Distrib. Sens. Netw..

[8] Xu G.B., Shen W.M., Wang X.B. (2014). Applications of Wireless Sensor Networks in Marine Environment Monitoring: A Survey. Sensors.

[9] Zhao Q., Li H., Li H., Wang H. Design of Power Management System Based on Wireless Sensor Node. Proceedings of the 2009 Symposium on Photonics and Optoelectronics.

[10] Zytoune O., Fakhri Y., Aboutajdine D. Lifetime Optimization for Wireless Sensor Networks. Proceedings of the 2009 IEEE/Acs International Conference on Computer Systems and Applications.

[11] Wang W., He Y., Zhang D., Wu Y., Pan D. (2020). Multi-Criteria Evaluation of Best Available Treatment Technology for Waste Lead-Acid Battery: The Case of China. Sustainability.

[12] Shen J., Li X., Shi X., Wang W., Zhou H., Wu J., Wang X., Li J. (2020). The toxicity of lithium to human cardiomyocytes. Environ. Sci. Eur..

[13] Moreno-Merino L., Jiménez-Hernández M.E., de la Losa A., Huerta-Muñoz V. (2015). Comparative assessment of button cells using a normalized index for potential pollution by heavy metals. Sci. Total Environ..

[14] Liu H., Hou C., Lin J., Li Y., Shi Q., Chen T., Sun L., Lee C. (2018). A non-resonant rotational electromagnetic energy harvester for low-frequency and irregular human motion. Appl. Phys. Lett..

[15] Liu H., Fu H., Sun L., Lee C., Yeatman E.M. (2020). Hybrid energy harvesting technology: From materials, structural design, system integration to applications. Renew. Sustain. Energy Rev..

[16] Agarwal M., Munjal A., Dusane R. (2018). To Demonstrate the Potential Application of “Low Temperature and High Performance Silicon Heterojunction Solar Cells Fabricated Using HWCVD” in Wireless Sensor Network: An Initial Research. J. Sol. Energy Eng. Trans. ASME.

[17] Horng G.J., Wu H.T. (2015). The Adaptive Path Selection Mechanism for Solar-Powered Wireless Sensor Networks. Wirel. Pers. Commun..

[18] Ibrahim R., Chung T.D., Hassan S.M., Bingi K., Salahuddin S. (2017). Solar Energy Harvester for Industrial Wireless Sensor Nodes. Procedia Comput. Sci..

[19] Liu D., Chen B., An J., Li C., Liu G., Shao J., Tang W., Zhang C., Wang Z.L. (2020). Wind-driven self-powered wireless environmental sensors for Internet of Things at long distance. Nano Energy.

[20] Zhang C., He X.F., Li S.Y., Cheng Y.Q., Rao Y. (2015). A Wind Energy Powered Wireless Temperature Sensor Node. Sensors.

[21] Tang M., Guan Q., Wu X., Zeng X., Zhang Z., Yuan Y. (2019). A high-efficiency multidirectional wind energy harvester based on impact effect for self-powered wireless sensors in the grid. Smart Mater. Struct..

[22] Tang M., Guan Q., Wu X., Zeng X., Zhang Z., Yuan Y. (2018). Wireless Sensor Network Utilizing Radio-Frequency Energy Harvesting for Smart Building Applications. IEEE Antennas Propag. Mag..

[23] Naderi M.Y., Chowdhury K.R., Basagni S., Heinzelman W., De S., Jana S. Surviving Wireless Energy Interference in RF-harvesting Sensor Networks: An Empirical Study. Proceedings of the 2014 Eleventh Annual IEEE International Conference on Sensing, Communication, and Networking Workshops.

[24] Zhu Y.H., Li E.T., Chi K.K. (2018). Encoding Scheme to Reduce Energy Consumption of Delivering Data in Radio Frequency Powered Battery-Free Wireless Sensor Networks. IEEE Trans. Veh. Technol..

[25] Wang L., Zhao L., Luo G., Zhao Y., Yang P., Jiang Z., Maeda R. (2020). System level design of wireless sensor node powered by piezoelectric vibration energy harvesting. Sens. Actuators A-Phys..

[26] Owen T.H., Kestermann S., Torah R., Beeby S.P. (2009). Self powered wireless sensors for condition monitoring applications. Sens. Rev..

[27] Zhang X., Fang J., Meng F., Wei X. (2014). A Novel Self-Powered Wireless Sensor Node Based on Energy Harvesting for Mechanical Vibration Monitoring. Math. Probl. Eng..

[28] Kapica J. (2020). Wind and photovoltaic potential in Europe in the context of mid-term energy storage. J. Renew. Sustain. Energy.

[29] Lehner M., Ascher A., Eberhardt M., Biebl E. A Solar Powered UHF Transponder for Wildlife and Low Light Applications. Proceedings of the 2015 IEEE 6th International Symposium on Microwave, Antenna, Propagation, and Emc Technologies.

[30] Ibrahim H.H., Singh M.S., Al-Bawri S.S., Islam M.T. (2020). Synthesis, Characterization and Development of Energy Harvesting Techniques Incorporated with Antennas: A Review Study. Sensors.

[31] Cao D., Ding X., Guo X., Yao M. (2020). Improved Flow-Induced Vibration Energy Harvester by Using Magnetic Force: An Experimental Study. Int. J. Precis. Eng. Manuf. -Green Technol..

[32] Nabavi S.F., Farshidianfar A., Afsharfard A. (2018). Novel piezoelectric-based ocean wave energy harvesting from offshore buoys. Appl. Ocean Res..

[33] Suhaimi K., Ramlan R., Putra A., Nor M.J.M., Nuawi M.Z., Abdullah S., Zulkifli R., Haris S.M., Nopiah Z.M., Arifin A. (2014). Translation to Rotary Frequency-up Conversion Vibration Based Energy Harvesting Device for Human Body Motion. Noise, Vibration and Comfort.

[34] Wei C.F., Jing X.J. (2017). A comprehensive review on vibration energy harvesting: Modelling and realization. Renew. Sustain. Energy Rev..

[35] Honma H., Mitsuya H., Hashiguchi G., Fujita H., Toshiyoshi H. (2018). Improvement of energy conversion effectiveness and maximum output power of electrostatic induction-type MEMS energy harvesters by using symmetric comb-electrode structures. J. Micromech. Microeng..

[36] Hammad B., Abdelmoula H., Abdel-Rahman E., Abdelkefi A. (2019). Nonlinear Analysis and Performance of Electret-Based Microcantilever Energy Harvesters. Energies.

[37] Li J., Tong X., Oxaal J., Liu Z., Hella M., Borca-Tasciuc D.A. (2019). Investigation of parallel-connected MEMS electrostatic energy harvesters for enhancing output power over a wide frequency range. J. Micromech. Microeng..

[38] Park J., Lee S., Kwak B.M. (2012). Design optimization of piezoelectric energy harvester subject to tip excitation. J. Mech. Sci. Technol..

[39] Raju S.S., Umapathy M., Uma G. (2015). Cantilever piezoelectric energy harvester with multiple cavities. Smart Mater. Struct..

[40] Morimoto M., Tsujiura Y., Koshiba Y., Kanno I., Ishida K. (2017). Vibration energy harvester with piezoelectric properties using polyurea thin films. Mol. Cryst. Liq. Cryst..

[41] Fu H.L., Yeatman E.M. (2017). A methodology for low-speed broadband rotational energy harvesting using piezoelectric transduction and frequency up-conversion. Energy.

[42] Wu Y., Qiu J., Zhou S., Ji H., Chen Y., Li S. (2018). A piezoelectric spring pendulum oscillator used for multi-directional and ultra-low frequency vibration energy harvesting. Appl. Energy.

[43] Roy S., Podder P., Mallick D. (2016). Nonlinear Energy Harvesting Using Electromagnetic Transduction for Wide Bandwidth. IEEE Magn. Lett..

[44] Chen S.J., Wu J.Y. (2016). Fabrication of a 2-DOF electromagnetic energy harvester with in-phase vibrational bandwidth broadening. Smart Mater. Struct..

[45] Jo S.E., Kim M.S., Kim Y.J. (2012). Electromagnetic human vibration energy harvester comprising planar coils. Electron. Lett..

[46] Uluşan H., Yaşar O., Zorlu Ö., Külah H. (2015). Optimized Electromagnetic Harvester with a Non-Magnetic Inertial Mass. Procedia Eng..

[47] Foisal A.R.M., Chung G. (2012). Design and Analysis of a Vibration-driven AA Size Electromagnetic Energy Harvester Using Magnetic Spring. Trans. Electr. Electron. Mater..

[48] Geisler M., Boisseau S., Perez M., Gasnier P., Willemin J., Ait-Ali I., Perraud S. (2017). Human-motion energy harvester for autonomous body area sensors. Smart Mater. Struct..

[49] Bendame M., Abdel-Rahman E., Soliman M. (2016). Wideband, low-frequency springless vibration energy harvesters: Part I. J. Micromech. Microeng..

[50] Fan K., Cai M., Wang F., Tang L., Liang J., Wu Y., Qu H., Tan Q. (2019). A string-suspended and driven rotor for efficient ultra-low frequency mechanical energy harvesting. Energy Convers. Manag..

[51] Gu Y., Liu W., Zhao C., Wang P. (2020). A goblet-like non-linear electromagnetic generator for planar multidirectional vibration energy harvesting. Appl. Energy.

[52] Xu J., Tang J. (2015). Multi-directional energy harvesting by piezoelectric cantilever-pendulum with internal resonance. Appl. Phys. Lett..

[53] Fu H., Theodossiades S., Gunn B., Abdallah I., Chatzi E. (2020). Ultra-low frequency energy harvesting using bi-stability and rotary-translational motion in a magnet-tethered oscillator. Nonlinear Dyn..

[54] Pillatsch P., Yeatman E.M., Holmes A.S. (2014). A piezoelectric frequency up-converting energy harvester with rotating proof mass for human body applications. Sens. Actuators A-Phys..

[55] Ramezanpour R., Nahvi H., Ziaei-Rad S. (2016). Electromechanical behavior of a pendulum-based piezoelectric frequency up-converting energy harvester. J. Sound Vib..

[56] Lee B.C., Chung G.S. (2016). Design and analysis of a pendulum-based electromagnetic energy harvester using antiphase motion. IET Renew. Power Gener..

